# Cognitive and Affective-Motivational Factors as Predictors of Students’ Home Learning During the School Lockdown

**DOI:** 10.3389/fpsyg.2021.751120

**Published:** 2021-12-08

**Authors:** Kathrin Lockl, Manja Attig, Lena Nusser, Ilka Wolter

**Affiliations:** Department Competencies, Personality, Learning Environments, Leibniz Institute for Educational Trajectories, Bamberg, Germany

**Keywords:** home learning, COVID-19, longitudinal, motivation, competencies

## Abstract

During the COVID-19 pandemic, students were facing great challenges. Learning was shifted from the classroom to the home of the students. This implied that students had to complete their tasks in a more autonomous way than during regular lessons. As students’ ability to handle such challenges might depend on certain cognitive and motivational prerequisites as well as individual learning conditions, the present study investigates students’ cognitive competencies as well as affective-motivational factors as possible predictors of coping with this new learning situation at home. The study uses data of Starting Cohort 2 of the German National Educational Panel Study (NEPS). Data of two measurement points are analyzed: Predictors were assessed at the earlier time point, when students (*N*=1,452; *M*_age_=12years, 8months; 53.4% female) mostly attended seventh grade of a secondary school. They completed competence tests in reading as well as mathematics and rated affective-motivational aspects in terms of willingness to exert effort, learning enjoyment, and intrinsic motivation. One and a half years later − during the COVID-19 pandemic and the first period of school closures − the second measurement point took place. Students’ parents rated the situation of learning at home with respect to students’ coping with the new situation and parents’ difficulties to motivate them. Regression analyses controlling for school track, students’ gender, and parents’ educational level and parental stress revealed that students’ reading competencies and their willingness to exert effort were significant predictors of their coping with the new learning situation at home. Moreover, parents reported that boys were more difficult to motivate to learn during this time as compared to girls. Other predictors (e.g., learning enjoyment) turned out to be non-significant when entered simultaneously in the regression analyses. The results point to the importance of children’s prerequisites for autonomous learning situations without structuring elements by teachers within the school context.

## Introduction

During the COVID-19 pandemic, enormous challenges in all life domains emerged. They affected not only people’s work, leisure time, and family life but also educational processes in formal and non-formal learning environments. In order to prevent the spread of SARS-Cov-2, educational institutions like childcare facilities, schools, and universities closed and shifted learning from the classroom to the homes of the students. That is, home teaching was implemented as an alternative to classroom teaching. This shift implied that students had to complete their tasks in a more autonomous way without or with less educational assistance and teacher support than during regular lessons at school. The challenges were particularly wearing during the first school closures in spring 2020 when teachers and students were forced to adjust to distance teaching with little to no time for preparation (for an overview regarding the situation in Germany, Austria and Switzerland in spring 2020 see Helm et al., 2021).

As surveys showed (Huber et al., 2020; Letzel et al., 2020), one of the greatest challenges for students themselves was taking over more responsibility for their own learning activities and this seems to have been associated with certain difficulties. For example, during this time, students stated to have problems with initiating their daily learning sessions, concentrating on the homework, and structuring their day (Huber et al., 2020). Letzel et al. (2020) reported that there was little contact between teachers and their students and that about 50% of students would have liked more support and feedback from their teachers. These examples suggest that the structure provided by teachers was much less emphasized during that time compared to regular instruction during school hours. In this novel situation with little external structure, students had to invest more in self-structuring and self-organizing of their daily schedule. In this context, students’ cognitive and affective-motivational prerequisites to coping with home schooling are expected to be relevant. The present study therefore investigates the role of such prior student prerequisites on their coping with home learning during the first school closures in Germany. In doing so, we focus on two components of coping in this situation: a cognitive component of coping with the demands of home learning as well as an affective-motivational component of being motivated to engage in learning activities during school closures.

From a scientific perspective, home learning during the Covid-19 pandemic is still a relatively new situation. Therefore, there is no explicit or comprehensive theoretical framework that addresses students’ learning during the school closures. However, some theoretical approaches and empirical findings related to learning in general may help to shed light on the mechanisms that foster students’ autonomous learning. For instance, research on instructional quality (Klieme et al., 2009), on self-regulated learning (Zimmermann, 2001), on homeschooling (Ray, 2020), or on distance education (Simonson et al., 2011) suggests a theoretical background to examine different aspects related to home learning as well. Another line of related research refers to research on homework (Trautwein et al., 2006). Although framed to explain how students deal with work assignments that are rather limited in time and that are meant to be carried out during non-school hours, research on homework is especially informative when it comes to the impact of several student prerequisites (e.g., cognitive and affective-motivational factors) on learning behavior. Against this background, in the following sections, we first review work on cognitive prerequisites that might be relevant to deal with home learning before turning to affective-motivational factors.

## Predictors of Home Learning

### Cognitive Prerequisites

Cognitive prerequisites refer to cognitive bases that are necessary for learning and that can influence how students are able to cope with home learning and how they engage in learning situations in different ways. For instance, prior knowledge provides a structure into which new information can be encoded and from which it can be retrieved later (e.g., Ericsson and Kintsch, 1995). Therefore, it supports the acquisition of new knowledge and helps in future learning (Hambrick, 2003). However, prior achievement and general cognitive abilities do not only affect further achievement (Anderson et al., 1989; Slavin et al., 1990) but also students’ behavioral engagement (e.g., Chen et al., 2013; Garon-Carrier et al., 2016). Accordingly, research demonstrated effects of general cognitive abilities and prior academic achievement on behavioral homework engagement (Trautwein et al., 2002; Trautwein and Köller, 2003; Rodriguez et al., 2019).

Moreover, cognitive prerequisites may also influence students’ engagement in learning situations in an indirect way. That is, higher cognitive abilities and prior academic achievement have been shown to exert a positive effect on motivation (Valentine and Dubois, 2005; Trautwein et al., 2006; Schöber et al., 2018) probably because students with high cognitive abilities are more confident of being able to complete the assignments (Trautwein et al., 2006).

Most of the studies that investigated the contribution of cognitive prerequisites to an engaged learning behavior used relatively broad and unspecific measures of cognitive functioning. Furthermore, these measures were often used in the sense of control variables. In order to investigate achievement gains, some studies included domain-specific achievement tests (e.g., in the area mathematics, Trautwein et al., 2002). However, to our knowledge, no studies have yet examined whether domain-specific competencies support students’ learning engagement across different school subjects.

The relevance of domain-specific competencies (e.g., reading and mathematical competencies) has been emphasized by international large-scale assessments of students’ performance (e.g., Programme for International Student Assessment (PISA); OECD, 2016). These basic competencies measured in different application situations are subject to domain-specific development in the early years but can be conceptualized along the lifespan as cross-subject basic competencies (Weinert et al., 2019). Reading competence is not only important in the school subject “German” (or other language arts), but also for coping with the educational requirements in other domains. The range of reading occasions is very wide and reading fulfills different functions at the same time (*cf*. Groeben and Hurrelmann, 2004). Especially in the situation of home learning, the ability to understand written texts may be an essential prerequisite for being able to complete the written assignments.

Besides reading, mathematical literacy is an important key competence since the requirement to understand and apply mathematical data and methods in a variety of situations is becoming increasingly important (Mullis et al., 2012). Mathematical competencies are necessary in many different settings where calculations must be made, mathematical or abstract problems must be solved, or different representations of numbers must be understood. Therefore, it may also be relevant for other school subjects, e.g., mathematical concepts and procedures are necessary to solve problems in science or economics.

Based on these assumptions highlighting the importance of reading and mathematical competencies, the present study includes both competencies as prerequisites to predict students dealing with the home learning situation during the pandemic-related school closures.

### Affective-Motivational Prerequisites

Moreover, students’ learning (behavior) also depends on affective and motivational factors (Steinmayr and Spinath, 2009; Murayama et al., 2013). The affective-motivational aspects include emotions related to a specific situation or interest and motivation related to a task or subject matter. Therefore, those can be the value connected or assigned to a subject or task, the experienced enjoyment, or the intrinsic motivation to engage in this task or situation. Previous research showed that enjoyment eventually contributes to students’ intrinsic motivation (*cf*. Ryan and Deci, 2000a), yet it is considered an emotion and as such also a strong indicator of students’ sustainable effort and persistence in learning. In comparison, intrinsic motivation should be mostly initiated by interest and the way in which students’ attention and exploratory behavior are directed. In comparison to test anxiety as a negative emotion related to achievement outcomes (e.g., Elliot and McGregor, 1999; Steinmayr et al., 2016), positive emotions, such as learning enjoyment, are less often in the focus of research on learning outcomes, even though previous research highlights the impact of positive emotions on the achievement of students (e.g., Pekrun et al., 2002). To this end, both positive emotions, such as learning enjoyment, as well as motivational aspects, such as the intrinsic motivation, might have affected students’ learning efforts during school closures in addition to their willingness to exert effort. Affective-motivational factors are most relevant predictors of human behavior (McAdams and Olson, 2010) and students with more positive emotions have been found to show higher achievement gains over time (Stipek et al., 2010).

During the school closures due to the COVID-19 pandemic, students’ affective and motivational prerequisites may have been even more important than during normal school times. In school, teachers support children’s learning by structuring lessons, e.g., through instruction, individual assignments, or group work. In contrast, when there is no teacher present, students are responsible for regulating their own learning process. Therefore, self-regulated learning (Corno, 1994; Zimmermann, 2001; Boekaerts, 1999; Brunstein and Spörer, 2001) and motivational components (e.g., intrinsic motivation), which are assumed to influence self-regulated learning (Boekaerts, 1999; see also Pintrich, 1999), are expected to be central factors in this self-organized learning situation.

In the present study, we focus on three aspects of students’ learning behavior (Domínguez et al., 2010) and that we assume to be particularly important to home learning: intrinsic motivation, willingness to exert effort, and learning enjoyment. First, as previous studies have shown, students’ motivation is positively associated with indicators of their learning behavior and predicts achievement outcomes beyond cognitive prerequisites (Steinmayr and Spinath, 2009). More motivated students show higher learning engagement, e.g., with regard to the time spent on homework (Dettmers et al., 2009; Regueiro et al., 2015), the management of homework time (Núñez et al., 2015), and the amount of homework done (Regueiro et al., 2017). Moreover, research suggests that students’ engagement depends on the type of motivation for a task (Ryan and Deci, 2000b). In particular, intrinsic motivation is considered a beneficial precondition since students who work on tasks driven by intrinsic reasons more likely show high levels of persistence, achievement, and engagement (Flink et al., 1992; Bouffard et al., 2001; Coutts, 2004).

Second, willingness to make an effort is a very relevant coping strategy of staying persistent when facing challenges (Moore et al., 2015). Persistent learning behavior at the beginning of primary school even predicts academic outcomes of achievement and college graduation several years later (McClelland et al., 2013). Persistence in task engagement and in learning also has as a mediating effect between the quality of the learning environment and academic achievement (Domínguez et al., 2010; Schmerse, 2020). This is especially relevant with respect to the novel situation of learning at home and an uncertain quality of learning offers and instruction. Moreover, willingness to exert effort is related to consciousness (Vasalampi et al., 2014) which has been found to have positive effects on educational engagement and attainment (De Raad and Schouwenburg, 1996).

Finally, enjoyment in learning represents a positive emotion toward learning requirements that may occur during task completion (Pekrun et al., 2002). Various studies have demonstrated that students who enjoy learning tend to exert more effort and to persist for longer even when they struggle with difficult tasks, which results in an increase in their success rates (Yaratan and Kasapoğlu, 2012; García et al., 2016). The associations between learning enjoyment and positive learning behavior as being more engaged (Reschly et al., 2008) and being more persistent (Gendolla, 2003) leads to more academic progress over time (Hagenauer and Hascher, 2014). Thus, intrinsic motivation, willingness to exert effort, and learning enjoyment represent mechanisms to explain learning success over time (Moore et al., 2015).

## Research Questions

Against this background, the present study focuses on how students managed the novel situation of learning at home during the first school closure in spring 2020. Based on previous research, it is plausible to assume that cognitive prerequisites and affective-motivational factors shape learning behavior – even when learning takes place under new circumstances as it was the case during school closures.

With respect to the dependent variables, managing the situation of relatively self-organized learning at home includes two different components: A cognitive component that refers to cognitively being able to understand instructions and master the requirements of the tasks as well as an affective-motivational component that involves being engaged in learning activities and staying focused on the tasks.

Thus, we were interested in how cognitive and affective-motivational factors contributed to successful coping with and sufficient motivation to engage in learning activities during that time. Therefore, we examined if prior cognitive competencies (reading and mathematical competencies) and affective-motivational factors (willingness to exert efforts, learning enjoyment, and intrinsic motivation) predict how well students cope with home learning during the pandemic-related school closures and how difficult it was to motivate them for home learning. In addition, we aimed at evaluating possible differential effects of the predictors on the outcome measures. That is, from a theoretical perspective, it could be argued that cognitive competencies might be more relevant for the cognitive aspect of coping with home learning, whereas affective-motivational factors could be assumed to be more important for students’ motivation in this situation.

## Materials and Methods

In the present study, we analyzed data from Starting Cohort 2 of the longitudinal German National Educational Panel Study (NEPS; Blossfeld et al., 2011). Starting Cohort 2 was initiated in 2010 with kindergarten children throughout Germany. With the beginning of elementary school, additional classmates were recruited and included in the sample. Participation was voluntarily. Parents gave informed consent for themselves and/or their child to participate in the study. Both children and their parents have been continuously followed since then (*n*=9,337). The annual survey program includes parental and child interviews on educationally relevant constructs (e.g., resources at home, parental engagement, and motivation) and family background as well as competence testing in various domains. Due to the COVID-19 pandemic in spring 2020, all parents were invited to an additional online survey. It covered relevant characteristics of learning at home during school closure (including students’ coping with learning at home and motivational difficulties).

### Sample

The analytic sample comprises *N*=1,452 students whose parents participated in the additional online survey during the COVID-19 pandemic in spring 2020. In the sample, 53.4% of the students were female and students’ mean age was 14years and 2months (range=12years and 7months to 15years and 5months, *SD*=4.2months). Whereas 66.6% of the students attended the highest school track (i.e., Gymnasium) at the start of the 2019/2020 school year, 33.4% of the students attended other school tracks. Furthermore, 61.3% of the families had an academic background (with at least one parent having a university degree), whereas 38.7% of the families had a non-academic educational background. Families’ highest international socioeconomic status (HISEI; see Ganzeboom et al., 1992; Ganzeboom, 2010) that combines income and education to indicate the status of an occupation was on average 64.7 (*SD*=15.4). The lowest value of the index is 16 (e.g., support staff and cleaners), the highest 90 (judges). In comparison, the mean HISEI of students in Germany in the year 2008 was 47.6 (Nold, 2010). These data suggest that a relatively high proportion of higher educated parents participated in the additional survey of NEPS. In the analyses, we used sample weights to deal with this bias in the sample composition (see below). More information about NEPS, Starting Cohort 2, and the NEPS-C additional online survey can be found online at https://www.neps-data.de.

### Measures

Our analyses draw on two measurement points of Starting Cohort 2. The earlier measurement point took place in fall 2018 when students were attending Grade 7. At this time point, all predictor variables were assessed including students’ competence in reading and mathematics as well as affective-motivational aspects in terms of willingness to exert effort, learning enjoyment, and intrinsic motivation. The later measurement point refers to the aforementioned additional online survey and covered outcomes including relevant characteristics of learning at home during school closure (including students’ coping with learning at home and motivational difficulties).

#### Predictor Variables

##### Cognitive Competence Measures

The competence tests were administered by trained interviewers as paper-pencil tests in individual sessions at students’ homes. The test duration was 28min each for the reading and mathematical competence test. Please note that due to time constraints in the survey, a randomly assigned rotation design was applied which resulted in a reduced number of students with data in the competence tests (*n*=798 for reading; *n*=843 for mathematics). The sequence of the tests was also randomly assigned to all study participants.

###### Reading Competence

The framework for the assessment of reading competence in the NEPS considers different text functions as well as cognitive requirements of the test items to assess reading competence (Gehrer et al., 2013; Weinert et al., 2019). Reading comprehension was measured using five text functions, namely: (a) informational texts, (b) commenting or arguing texts, (c) literary texts, (d) instructional texts, and (e) advertising texts (Gehrer et al., 2013). The cognitive requirements of test items were finding information in the text, drawing text-related conclusions, and reflecting and assessing. Most of the tasks were designed using a multiple-choice answering format. The rest of the tasks used a decision-making or matching response format (Gehrer et al., 2013). The reading competence test in Grade 7 was administered in two different versions with varying degrees of difficulty and assigned to the students dependent on prior competence assessments in Grade 4. The unidimensional tests included either 29 or 30 items and showed good item fit. The reliability of the reading competence test was good with EAP/PV reliability=0.83 and WLE reliability=0.79 (Krannich et al., 2017).

###### Mathematical Competence

The underlying framework of the tests on mathematical competence used in the NEPS combines mathematical content areas with mathematical and cognitive processes (see Neumann et al., 2013). The content areas refer to (a) quantity, (b) space and shape, (c) change and relationships, and (d) data and chance. The six cognitive processes refer to mathematical communication, mathematical argumentation, and modeling, using representational forms, mathematical problem solving as well as technical abilities and skills. Similar to the reading competence tests, there were two levels of difficulty which were assigned according to the prior competence level in Grade 4. Both mathematics tests consisted of 21 items requiring either multiple-choice or short constructed responses. The test showed good item fit and good reliability (EAP/PV reliability=0.77, WLE reliability=0.74; Kock et al., 2021).

Both tests were scaled using models of the item response theory (Pohl and Carstensen, 2012) and weighted maximum likelihood estimates (WLEs) were used in the analyses.

##### Affective-Motivational Factors

The survey items were integrated in a student questionnaire that could be completed either as a paper version or as an online version. Items on the child’s affective-motivational factors were taken from the German questionnaire on emotional and social school experiences of children (Rauer and Schuck, 2003) and adapted from Schiefele et al. (2002).

###### Intrinsic Motivation

Students’ intrinsic motivation was addressed with a scale containing four items, e.g., “I study for school because I enjoy working with the subject matter” and “I study for school because I think the contents are relevant” (Schiefele et al., 2002). The rating scale ranged from 1 (does not apply at all) to 4 (does completely apply) and had a good internal consistency (Cronbach’s alpha) with *α*=0.83.

###### Willingness to Exert Effort

This scale focuses on students’ willingness to exert effort especially when faced with great challenges and difficult tasks. It includes four items, e.g., “I try hard even when tasks are difficult” and “I complete all tasks with great accuracy.” The items were rated on a 4-point-Likert scale ranging from 1 (completely disagree) to 4 (completely agree). Internal consistency (Cronbach’s alpha) of this scale was acceptable (*α*=0.62).

###### Learning Enjoyment

The scale on learning enjoyment refers to students’ positive attitudes toward school and learning and includes three items, e.g., “I like attending school” and “I really enjoy learning at school.” Again, the items were rated on a 4-point Likert scale ranging from 1 (completely disagree) to 4 (completely agree). The scale on learning enjoyment showed a good internal consistency (Cronbach’s alpha) with *α*=0.89.

For all three scales, mean values were used in the following analyses.

#### Outcome Variables

The variables assessed at the second measurement point were based on parents’ ratings about their children’s learning at home during school closure. In detail, the parents had to state to what extent the following statements applied to them: (1) “My child coped well with the demands of home learning.” and (2) “It was difficult to motivate my child to study at home.” Students’ coping with learning at home and their motivational difficulties were assessed on a 5-point-Likert scale ranging from 1 (does not apply at all) to 5 (does completely apply). In the analyses, each item was treated as a distinct variable.

#### Control Variables

Additionally, we included several variables which might be correlated with the predictor and outcome variables. Control variables were the education of the parents, which was assessed with the International Standard Classification of Education (ISCED; Schroedter et al., 2006), students’ gender and the school type they attended at the beginning of the school year 2019/2020. Furthermore, we controlled for parents’ perception of their own stress and burden during the COVID-19 pandemic because this might have influenced their assessment of their children’s dealing with the home learning situation. To assess their stress and burden, parents had to indicate on a 5-point-Likert scale to what extent the following statements applied to them: “I was very stressed by the school closure and the demands of home schooling.”

### Analyses Plan

In order to investigate whether students’ prior cognitive competencies and affective-motivational factors were significant predictors of their learning at home, we computed separate regression analyses predicting parents’ ratings on (a) children’s coping with home learning and (b) the difficulty to motivate them. As we aimed to evaluate the relative and incremental impact of cognitive competencies and affective-motivational factors, we applied a stepwise procedure. In the first step (model 1), only the background variables including the highest education level of the parents (HISCED), the school track the students were attending, their gender and the level of stress that parents perceived during the COVID-19 pandemic were considered. To investigate whether cognitive competencies (model 2) or affective-motivational factors (model 3) additionally contributed to the prediction of the outcome variables, these indicators were added next. Finally, all variables were entered to investigate whether students’ cognitive competencies and affective-motivational factors accounted for separate or overlapping variance in explaining students’ coping with learning at home.

The regression analyses were carried out using MPlus version 7.4 (Muthén and Muthén, 2015). To deal with missing data, we applied the full information maximum likelihood (FIML) approach (e.g., Arbuckle, 1996) that is implemented in MPlus and uses valid information of all observations for model estimation. FIML has been shown to be superior to other missing data strategies (e.g., listwise deletion or mean replacement) and to provide more accurate estimates of regression coefficients and variance accounted (Enders, 2001). To support the FIML approach and to offer a broader data basis to deal with missing data, we included children’s cognitive competencies assessed at Grade 4 and affective-motivational factors (willingness to exert effort, learning enjoyment, and intrinsic motivation) measured at Grades 4, 5, and 6 as auxiliary variables.

In order to account for the biased sample composition, sample weights were used in the analyses (Würbach et al., 2021). Because the data were weighted and post-stratified in the analyses, statements can be generalized.

## Results

In the following sections, we first report descriptive results regarding the predictor variables assessed in Grade 7 and the outcome measures assessed one and a half years later. Moreover, the intercorrelations among these measures are presented. Finally, we show the results of multiple regression analyses to examine the contribution of each predictor variable on the outcome variables.

### Preliminary Analyses of Predictor and Outcome Variables

A summary of the descriptive statistics for children’s cognitive competencies, their affective-motivational factors, and parents’ rating of the home learning situation is displayed in Table 1. Reading and mathematical competencies were estimated as weighted maximum likelihood estimates (Pohl and Carstensen, 2012) and, as a result of the scaling, the corresponding mean scores are approximately zero. Furthermore, on average students reported their willingness to exert effort to be relatively high (*M*=3.02; *SD*=0.50). In comparison, they estimated their enjoyment of learning (*M*=2.65; *SD*=0.77) and their intrinsic motivation (*M*=2.42; *SD*=0.62) to be somewhat lower. Parents’ judgments concerning their children’s coping with home learning during the COVID-19 pandemic had a mean value of *M*=3.66 (*SD*=1.04) which was in the upper range of the scale. Furthermore, there was considerable variance with regard to parents’ rating on how difficult it was to motivate their children (*M*=3.03; *SD*=1.33). Finally, parents perceived stress had a mean value of *M*=2.76 (*SD*=1.30), which is near the middle of the scale, and a quite large variance as well.

**Table 1:** Descriptives of the predictor and outcome variables.

	*N*	Min	Max	*M*	*SD*
Reading competence	798	–	–	−0.10	1.51
Mathematical competence	843	–	–	−0.25	1.32
Willingness to exert effort	1,249	1	4	3.02	0.50
Enjoyment of learning	1,246	1	4	2.65	0.77
Intrinsic motivation	1,246	1	4	2.42	0.62
Coping with home learning	1,452	1	5	3.66	1.04
Difficult to motivate	1,452	1	5	3.03	1.33
Parental stress	1,452	1	5	2.76	1.30

*Descriptives are based on sample weights*.

Table 2 shows the intercorrelations among the variables included in the study. First, as expected, there was a high correlation between students’ reading and mathematical competencies (*r*=0.66; *p*<0.001). Similarly, moderate to high correlations emerged among the different aspects concerning students’ affective-motivational factors, i.e., their willingness to exert effort, their enjoyment of learning, and their intrinsic motivation (*r*=0.44 to *r*=0.70; all *p*<0.001). More interestingly, parents’ ratings regarding their children’s coping with the home learning were positively associated with all indicators of children’s cognitive competencies and affective-motivational factors assessed one and a half years earlier (*r*=0.14 to *r*=0.26; all *p*<0.001). In contrast, there were negative correlations among parents’ ratings concerning the difficulty to motivate their children and indicators of children’s cognitive competencies and affective-motivational factors (*r*=−0.15 to *r*=−0.28; all *p*<0.001). Furthermore, as expected, the two ratings concerning the situation of learning at home (i.e., students’ coping with home learning and parents’ difficulties to motivate them) were negatively associated with each other (*r*=−0.54; *p*<0.001).

**Table 2:** Pearson correlations of the predictor, outcome, and control variables.

	1	2	3	4	5	6	7	8	9	10	11
1. Reading Competence	–	0.66^**^	0.13^**^	0.14^**^	0.17^**^	0.21^**^	−0.28^**^	0.29^**^	0.03	0.40^**^	−0.10^**^
2. Mathematical Competence	0.61	–	0.34^**^	0.08^**^	0.30^**^	0.23^**^	−0.15^**^	0.12^**^	−0.17^**^	0.42^**^	−0.16^**^
3. Willingness to exert effort	0.09	0.24	–	0.44^**^	0.54^**^	0.26^**^	−0.26^**^	−0.12^**^	0.08^**^	0.20^**^	−0.14^**^
4. Enjoyment of learning	0.13	0.08	0.43	–	0.70^**^	0.14^**^	−0.24^**^	−0.16^**^	0.25^**^	0.14^**^	−0.29^**^
5. Intrinsic motivation	0.12	0.21	0.54	0.70	–	0.19^**^	−0.18^**^	−0.16^**^	0.11^**^	0.26^**^	−0.19^**^
6. Coping with home learning	0.24	0.19	0.27	0.15	0.19	–	−0.54^**^	−0.02	0.11^**^	0.17^**^	−0.27^**^
7. Difficult to motivate	−0.29	−0.15	−0.30	−0.23	−0.20	−0.54	–	0.32	−0.23^**^	−0.08^**^	0.21^**^
8. Educ. background (HISCED)	0.28	0.16	−0.13	−0.12	−0.14	−0.00	0.03	–	−0.04	0.00	−0.04
9. Gender	0.09	−0.18	0.04	0.23	0.09	0.11	−0.23	−0.05	–	−0.08^**^	−0.01
10. School type	0.38	0.42	0.20	0.17	0.29	0.16	−0.08	0.03	−0.07	–	−0.14^**^
11. Parental stress	−0.08	−0.17	−0.12	−0.28	−0.17	−0.27	0.21	−0.05	−0.01	−0.11	–

*Intercorrelations based on the available data are presented above the diagonal, estimated intercorrelations based on the FIML are presented below the diagonal; all intercorrelations were calculated using sample weights (no significance level is displayed for the estimated intercorrelations)*.

^*^p<0.05,

** *p<0.01*.

Next, we tested whether the correlations of different predictor variables with a particular outcome variable were significantly different from each other. For instance, for coping with home learning, we examined whether the correlation of reading competence with coping (*r*=0.21) differed significantly from the correlation of mathematical competence with coping (*r*=0.23). The calculation according to Eid et al. (2011) (single-sided test) showed that there was no significant difference between these two correlations (*z*=0.48; *p*=0.32). However, the correlation of willingness to exert effort with coping (*r*=0.26) was significantly larger than the correlation of enjoyment of learning and coping (*r*=0.14; z=3.79; *p*<0.001), and the correlation of intrinsic motivation and coping (*r*=0.19; z=2.65; *p*=0.004). Moreover, there was a significant difference between the correlations of enjoyment of learning with coping and intrinsic motivation with coping (*z*=−1.88; *p*=0.03).

For the outcome variable difficulty to motivate, the correlation between reading competence and parents’ difficulty in motivating their children (*r*=−0.28) was significantly larger than the correlation between mathematical competence and parents’ difficulty in motivating their children (*r*=−0.15; z=2.81; *p*=0.002). Furthermore, the correlation of willingness to exert efforts with motivation difficulties (*r*=−0.26) was significantly larger than the correlation of intrinsic motivation and motivation difficulties (*r*=−0.18; z=2.83; *p*=0.002). There was also a significant difference between the correlations of enjoyment of learning with motivation difficulties (*r*=−0.24) and intrinsic motivation and motivation difficulties (*r*=−0.18; z=−2.91; *p*=0.002). Finally, the correlations of willingness to exert efforts with motivation difficulties and the correlation of learning enjoyment and motivation difficulties did not significantly differ from each other (z=0.43; *p*=0.34).

### Prediction of Students’ Learning at Home

As a next step, we computed regression analyses predicting parents’ ratings on children’s coping with home learning (see Table 3). The analysis in model 1 revealed that students’ school type (*β*=0.14; *p*=0.03) was a significant predictor, whereas parents’ educational level (*β*=−0.02; *p*=0.76) did not significantly explain variance of students’ coping with learning at home. There was no gender difference (*β*=0.12; *p*=0.054) in students’ coping with the situation during the pandemic-related school closures. Finally, parents’ perceived stress during the COVID-19 pandemic (*β*=−0.25; *p*<0.001) was negatively correlated with their ratings of how well their children were coping. In model 2, students’ cognitive competencies were added as predictors but neither reading competencies nor mathematical competencies significantly contributed to the prediction of the students’ coping. As shown in Table 3 (model 2), the effect of school type diminished when students’ cognitive competencies were included, suggesting a covariation of these variables. Model 3, which includes affective-motivational factors instead of cognitive competencies, revealed that students’ willingness to exert effort (*β*=0.22; *p*<0.001) was relevant for students’ coping with home learning, whereas their enjoyment of learning (*β*=−0.11; *p*=0.24) and their intrinsic motivation (*β*=0.07; *p*=0.43) did not significantly explain variance in this outcome variable. Further, there was a gender difference when including affective-motivational prerequisites in that girls were better able to cope with the situation of home learning (*β*=0.13; *p*=0.047). Moreover, parental stress was a significant predictor of students’ coping during school closures (*β*=−0.25; *p*<0.001). Finally, the analysis in model 4 with all variables showed that willingness to exert effort (*β*=0.23; *p*<0.001) remained to be a significant predictor when students’ cognitive competencies and affective-motivational factors were added simultaneously. Interestingly, the impact of students’ reading competencies (*β*=0.21; *p*=0.028) on their coping with home learning also became significant in the final and complete model 4. Regarding the control variables, only parental stress was a significant predictor, whereas there were no differences regarding gender and school type.

**Table 3:** Regression analyses predicting “Coping with Home Learning.”

	Model 1			Model 2			Model 3			Model 4			*b*	*SE*	*ß*	*b*	*SE*	*ß*	*b*	*SE*	*ß*	*b*	*SE*	*ß*
Intercept	*3.79*			*3.96*			*2.52*			*2.76*		
Background variables												
HISCED (Ref. no acad. background)	−0.04	0.13	−0.02	−0.15	0.13	−0.07	0.03	0.12	0.01	−0.10	0.12	−0.04
School (Ref. no Gymnasium)	0.30	0.14	0.14^*^	0.13	0.15	0.06	0.20	0.14	0.10	0.06	0.14	0.03
Gender (Ref. male)	0.25	0.13	0.12	0.22	0.15	0.10	0.27	0.14	0.13^*^	0.20	0.15	0.10
Parental Stress	−0.26	0.07	−0.25^**^	−0.25	0.07	−0.24^**^	−0.26	0.07	−0.25^**^	−0.26	0.06	−0.25^**^
Cognitive competencies												
Reading				0.11	0.08	0.15				0.15	0.07	0.21^*^
Mathematics				0.04	0.08	0.05				−0.03	0.08	−0.03
Affective-motivational factors												
Willingness to exert efforts							0.45	0.12	0.22^**^	0.47	0.12	0.23^**^
Enjoyment of learning							−0.15	0.12	−0.11	−0.17	0.12	−0.13
Intrinsic motivation							0.11	0.14	0.07	0.12	0.14	0.07
	*R*^2^ =0.10			*R*^2^ =0.13			*R*^2^ =0.15			*R*^2^ =0.18		

* *p<0.05*,

** *p<0.01*.

Overall, the amount of variance in the outcome measure explained by the predictor variables was rather low for model 1 with *R*^2^=0.10 but increased to *R*^2^=0.18 in model 4 when all variables were entered. This increase in the amount of explained variance suggests that cognitive competencies and affective-motivational factors independently contributed to the prediction of students’ coping with home learning.

Table 4 shows the results of the regression analyses predicting parents’ difficulty in motivating their children. Regarding the background variables in model 1, a negative correlation was found between students’ gender and the difficulty in motivating them (*β*=− 0.24; *p*=0.001). Meaning that, parents reported to have less difficulties to motivate girls as opposed to boys. Moreover, parents’ perceived stress during the COVID-19 pandemic (*β*=0.20; *p*=0.001) was positively correlated with their ratings of how difficult it was to motivate their children. The educational level of the parents (*β*=0.03; *p*=0.68) and the school type (*β*=−0.17; *p*=0.35) did not significantly explain variance in this outcome variable. When students’ cognitive competencies were added in model 2, the analyses revealed that students’ reading competencies were (*β*=−0.28; *p*=0.003) significant predictors of the difficulty to motivate them. Parents reported to have more difficulties to motivate their children when their reading competencies were lower. In contrast, the mathematical competencies of the students (*β*=−0.03; *p*=0.76) did not significantly predict motivation problems reported by parents. Similar to students’ coping with learning at home, model 3 showed that students’ willingness to exert effort (*β*=−0.26; *p*=0.004) significantly predicted parents’ judgments on motivation problems, whereas students’ enjoyment of learning (*β*=−0.04; *p*=0.63) and their intrinsic motivation (*β*=0.03; *p*=0.79) did not contribute to the prediction of motivation problems. In model 4, which included all predictor variables, the results remained the same. Students’ willingness to exert effort (*β*=−0.26; *p*=0.002), their reading competence (*β*=−0.31; *p*=0.001) as well as their gender (*β*=−0.18; *p*=0.014) and parents’ perceived stress (*β*=0.18; *p*=0.002) were significant predictors of motivation problems reported by parents. The other predictor variables did not explain additional variance in this outcome variable.

**Table 4:** Regression analyses predicting “Difficulty to Motivate.”

	Model 1			Model 2			Model 3			Model 4			*b*	*SE*	*ß*	*b*	*SE*	*ß*	*b*	*SE*	*ß*	*b*	*SE*	*ß*
Intercept	*3.39*			*3.00*			*5.47*			*4.98*		
Background variables												
HISCED (Ref. no acad. background)	0.08	0.19	0.03	0.32	0.19	0.11	−0.03	0.18	−0.01	0.20	0.17	0.07
School (Ref. no Gymnasium)	−0.20	0.21	−0.07	0.12	0.22	0.04	−0.07	0.19	−0.02	0.20	0.20	0.07
Gender (Ref. male)	−0.63	0.21	−0.24^**^	−0.54	0.20	−0.20^**^	−0.58	0.20	−0.22^**^	−0.47	0.20	−0.18^*^
Parental Stress	0.27	0.08	0.20^**^	0.26	0.08	0.19^**^	0.23	0.09	0.17^**^	0.24	0.08	0.18^**^
Cognitive competencies												
Reading				−0.26	0.09	−0.28^**^				−0.28	0.09	−0.31^**^
Mathematics				−0.03	0.10	−0.03				0.05	0.10	0.05
Affective-motivational factors												
Willingness to exert efforts							−0.67	0.23	−0.26^**^	−0.67	0.22	−0.26^**^
Enjoyment of learning							−0.07	0.15	−0.04	−0.04	0.15	−0.02
Intrinsic motivation							0.06	0.23	0.03	0.05	0.22	0.02
	*R*^2^ =0.11			*R*^2^ =0.17			*R*^2^ =0.17			*R*^2^ =0.23		

* *p<0.05*,

** *p<0.01*.

Again, the amount of variance in the outcome measure explained by the predictor variables increased when more variables were entered in the analyses *R*^2^=0.23, indicating that cognitive competencies and affective-motivational factors were equally important but independent predictors of parents’ perception of motivation problems during the pandemic-related school closures.

## Discussion

The present study focused on students’ situation of home learning during the first school closures due to the COVID-19 pandemic. Probably, the most important contribution of the study is that – due to the longitudinal nature of this study – it allows for a detailed inspection of cognitive and affective-motivational prerequisites that might have been important in dealing with the situation of home learning.

First, overall, parents reported in our study that their children were coping rather well with the new learning situation. This finding is in line with the results of a study by Wößmann et al. (2020) according to which parents’ assessment of the overall situation was rather positive. This result coincides with the perceptions of students themselves: More than half of the 15-year-old students in a study by Letzel et al. (2020) partly or fully agreed that, overall, they can handle home schooling very well. However, this rather positive impression should not conceal possible difficulties related to home learning. Our findings also showed that there was considerable variability with regard to parents’ ratings of their children’s motivation. About one third of parents (34%) reported that there were few to no difficulties motivating their children to learn at home. However, another third of the parents (35%) thought that their children were rather or quite difficult to motivate to learn. The remaining third (32%) were in the middle category. In a similar vein, Wildemann and Hosenfeld (2020) reported that around half of the parents of elementary and secondary school students stated that their children had low motivation or were not motivated at all. Furthermore, as also documented in the homework literature, the study revealed gender differences in motivation, with boys being more difficult to motivate than girls (Trautwein et al., 2006).

In addition, considering students’ prerequisites that were measured one and a half years before the pandemic revealed several interesting findings. Both cognitive and affective-motivational prerequisites of the students predicted students’ ability to handle the situation of home learning. More specifically, with regard to cognitive prerequisites, their reading competence turned out to be a significant predictor, whereas, with regard to affective-motivational factors, students’ willingness to exert efforts significantly predicted their coping of home learning and motivation. Contrary to our expectations, other cognitive (mathematics competence) and affective-motivational (enjoyment of learning and intrinsic motivation) prerequisites did not significantly contribute to the prediction of the dependent variables when entered simultaneously in the regression analyses.

Looking at cognitive competencies, reading competencies turned out to be a significant predictor not only for students’ coping of the new learning situation but also for their motivation during school closures as reported by their parents. Even though, the bivariate correlations of reading and mathematics competences with the two outcome variables were relatively low and differed only for motivation difficulties significantly from each other, the results of our study tentatively support the assumption that reading competencies can be considered as important basic cross-subject competencies (Weinert et al., 2019) that help to handle the situation of home learning. A cautious interpretation could be that compared to regular classroom instruction, dealing with written text materials, e.g., reading texts in schoolbooks or reading work instructions could have been of even greater relevance when students were studying at home. Studies showed that teachers often used online platforms or email as communication tools to distribute learning materials and work assignments (Wolter et al., 2020) during this first period of school closures in Germany and, at the same time, the opportunities for teachers to provide verbal instruction or give immediate feedback were substantially reduced. Experiencing comprehension difficulties may have resulted in lower confidence of being able to complete the assignments and in less positive expectancy beliefs (Trautwein et al., 2006), leading to lower motivation when working autonomously on the tasks.

In contrast to reading competencies, mathematical competencies were no significant predictors of students’ dealing with home learning when entered simultaneously in the regression analyses. However, when interpreting this finding, it has to be acknowledged that reading and mathematical competencies share common proportions of variance. Consistent with other findings, our results showed that mathematical and reading competencies were highly intercorrelated (e.g., Hooper et al., 2010). This correlation may be traced back to the fact that underlying abilities or skills, such as short-term memory, working memory, and executive functioning, are important preconditions for both reading and mathematical competencies (e.g., Alloway et al., 2006; Bull et al., 2008; Knievel et al., 2010). Therefore, these underlying abilities or skills may also have played an important role when students had to handle the situation of home learning. However, our results suggest there is no unique variance in the outcomes variables that could be explained by mathematical competencies when reading competencies were accounted for. One possible reason for this could be that the range of school subjects in which reading competencies were important, in particular in the case of rather self-organized home learning, was comparatively larger than for mathematical competencies.

With respect to the affective-motivational factors, willingness to exert effort predicted students’ behavior during school closures while intrinsic motivation and learning enjoyment were not relevant for their coping with the new learning situation. According to parents, students were more able to cope with the situation and showed fewer difficulties in motivation when students had higher levels of willingness to exert effort with respect to prior learning circumstances. Willingness to exert effort is especially relevant in challenging and demanding situations (Moore et al., 2015) and thus a crucial prerequisite in the novel times of school closures and learning at home when hardly any external structure was provided and the ability of students to regulate their own learning progress was particularly important. Based on our findings, willingness to exert effort might potentially serve as a relevant mediator of further motivational predictors leading to more engagement and ultimately to more successful coping strategies in learning (Reschly et al., 2008; Hagenauer and Hascher, 2014).

In contrast to our expectations and previous work (e.g., Trautwein et al., 2006; Hong et al., 2009; Xu, 2017; Suárez et al., 2019), students’ intrinsic motivation seemed to be less relevant in this learning situation at home. Similar to the findings regarding cognitive competencies, it could be considered that there is shared variance in the affective-motivational constructs that were included in the analyses. Nevertheless, our data indicated that in the special situation of home learning, the willingness to make an effort might have been more relevant compared to intrinsic motivation or learning enjoyment. Even though we cannot infer this directly from our data this could be due to the requirements in this novel situation, e.g., many tasks that had to be completed in a self-regulated way without sufficient support or immediate feedback provided by teachers. That fact that enjoyment in learning was not important in the present study might also be traced back to its operationalization. Items were closely related to learning at school and thus, maybe less related to learning activities at home. In fact, the learning situation at school and the one arising in spring 2020 were most likely not directly comparable.

In summary, the results revealed that children’s cognitive and affective-motivational factors independently contributed to how the situation of learning at home during school closures were experienced by parents. Although differential effects were expected against the theoretical background, the findings did not support the presumption that cognitive components were closer related to coping with the learning situation at home and that affective-motivational factors were more important for students’ motivation. Rather, both reading competence and willingness to exert effort simultaneously predicted both outcome variables, i.e., coping of learning at home and difficulties in motivation during school closures.

### Strength and Limitations

This study provides important findings on relevant predictors of coping with the special situation of home learning during pandemic-related school closures in Germany. Using the advantages of the large longitudinal sample of the NEPS, the present study considered data one and a half years prior to the pandemic regarding two competence domains of students as well as three affective-motivational factors. However, besides the manifold benefits of longitudinal data, we cannot rule out that especially the affective-motivational constructs were specific to the situation. Even though, results on earlier time points showed that there is moderate stability of these measures (with *r*’s between 0.50 and 0.60 over the course of 1year from Grade 6 to Grade 7), we cannot be sure of the stability of these measures in the face of the new situation which might have changed students’ willingness to exert effort, intrinsic motivation, and enjoyment of learning. Further, to increase representativeness of the present sample and to allow generalizable statements, sample weights were used. Yet overall, the amount of explained variance was relatively low with *R*^2^=0.18 for coping with home learning and *R*^2^=0.23 for motivation difficulties.

Nevertheless, some additional limitations should be noted. A limitation concerns the fact that the dependent variables were based on single items instead of using scales to measure students’ handling of home learning. Using a different way of operationalization not only would have allowed to compute standard reliability measures but also to measure a more heterogeneous composite of these constructs. At the time, the study was planned, the situation of home learning was both new and unexpected and therefore only a few findings from other studies were available (e.g., forsa, 2020). Therefore, it was barely possible to rely on existing scales on home learning and, consequently, the questions were designed for the current study to depict the situation and to address research questions from different research fields. Furthermore, instead of interviewing the students, the parents reported from their perspective on how their children were dealing with the situation of home learning. Consequently, it is not quite clear yet how students themselves perceived the situation. Although other studies showed that parents’ and children’s perceptions concerning home learning during the COVID-19 situation led to similar outcomes (Letzel et al., 2020; Wößmann et al., 2020), we might have obtained a better overall picture of the situation if it had been possible to include both perspectives. Later assessments of Starting Cohort 2 of the NEPS will help to address this limitation and will provide further information on how students themselves perceived the situation of learning from home.

Moreover, concerning the scales to measure affective-motivational factors it has to be noted that 4-point-Likert scales were used which were initially addressed to somewhat younger children in the course of this study. As the children have been participating in the longitudinal study since elementary school age, the scale was conducted without changes over time in order to maintain consistency. However, this may have led to restricted variance of these predictor variables.

Another limitation lies in the fact that due to the design of the study at the previous measurement point, there was a relatively high number of missing data in the reading and mathematical competence tests. In order to alleviate the problem, we used the FIML approach and added auxiliary variables from previous competence tests which should have provided a reliable basis for the estimation of the coefficients. Furthermore, it is important to mention that the missing values in the competence tests were missing at random and therefore, it can be assumed that the results were not biased in a specific way.

### Conclusion

To conclude, the present study extends previous research on the situation of home learning due to the COVID-19 pandemic by integrating prior cognitive and affective-motivational factors as predictors of students’ coping with home learning in secondary school in Germany. The findings suggest that in particular students’ reading competence and their willingness to exert effort contributed to their coping of home learning. Even though only tentative recommendations can be made on the basis of our study, it might be important to give special attention to comprehensible and less complex language in the text material and instructions that students have to work on in order to support students with lower reading competencies. Synchronous and interactive teaching elements in which students can approach teachers for questions might help clarifying problems of understanding. Moreover, the high variability that was found with regard to students’ motivation suggests that a relatively high proportion of students had problems to work on the tasks in a self-regulated way. Therefore, fostering self-regulation skills, e.g., by formulating concrete goals and expectations and by providing feedback might help to overcome these problems. Finally, more research is needed to investigate how children’s prerequisites influence their learning behavior and how interventions in the educational field could help to deal with challenging situations, such as home learning.

## Data Availability Statement

The present article analyzed data of the National Educational Panel Study in Germany. The anonymized data are available for the scientific community at: https://www.neps-data.de.

## Ethics Statement

The NEPS study is conducted under the supervision of the German Federal Commissioner for Data Protection and Freedom of Information (BfDI) and in coordination with the German Standing Conference of the Ministers of Education and Cultural Affairs (KMK) and – in the case of surveys at schools – the Educational Ministries of the respective Federal States. All data collection procedures, instruments, and documents were checked by the data protection unit of the Leibniz Institute for Educational Trajectories (LIfBi). The necessary steps are taken to protect participants’ confidentiality according to national and international regulations of data security. Participation in the NEPS study is voluntary and based on the informed consent of participants. This consent to participate in the NEPS study can be revoked at any time. All parents of the Starting Cohort 2 of the NEPS gave their written consent for themselves and their child for participation in the study. Written informed consent to participate in this study was provided by the participants’ legal guardian/next of kin.

## Author Contributions

All authors contributed to the conception and the design of the article and provided feedback and ideas. KL performed the statistical analysis. KL and LN took the lead in writing the manuscript. All authors contributed to the article and approved the submitted version.

## Funding

The authors have not received any funding for the publication of this article.

## Conflict of Interest

The authors declare that the research was conducted in the absence of any commercial or financial relationships that could be construed as a potential conflict of interest.

## Publisher’s Note

All claims expressed in this article are solely those of the authors and do not necessarily represent those of their affiliated organizations, or those of the publisher, the editors and the reviewers. Any product that may be evaluated in this article, or claim that may be made by its manufacturer, is not guaranteed or endorsed by the publisher.
